# Ultrafine grinding of wheat flour: Effect of flour/starch granule profiles and particle size distribution on falling number and pasting properties

**DOI:** 10.1002/fsn3.1431

**Published:** 2020-05-01

**Authors:** Erqi Guan, Yuling Yang, Jinyue Pang, Tingjing Zhang, Mengmeng Li, Ke Bian

**Affiliations:** ^1^ School of Food Science and Technology Henan University of Technology Zhengzhou China; ^2^ Henan Food Crop Collaborative Innovation Center Zhengzhou China

**Keywords:** damaged starch, particle size distribution, pasting profiles, superfine techniques, wheat flour

## Abstract

In the present paper, the properties of different ultrafine flour samples, including particle size distribution, damaged starch content, falling number, and pasting properties, were examined. The results indicated that the particle size decreased significantly after jet milling, as the rotation speed and grinding time increased, and the damaged starch content significantly increased as the size of the flour/starch decreased; this is in contrast to the significant decrease in falling number. Significant differences in pasting temperature were observed between straight‐grade flour (68.6°C) and five ultrafine flour samples (from 86.3 to 87.9°C). We also observed significant increases in peak viscosity, trough, breakdown viscosity, final viscosity, and setback as the flour particle size decreased from 43.07 µm to 25.81 µm (D_50_). The same parameters significantly decreased as the flour particle size decreased from 25.81 µm to 10.15 µm (D_50_). Correlation analysis identified a significant negative correlation between flour particle size (D_50_) and damaged starch content and pasting temperature, while a significant positive correlation was found with the falling number values. The results of this work may have an important impact on the quality of processed foods.

## Graphical abstract

The effects of ultrafine grinding on wheat flour/starch by jet milling was studied. Particle size and its distribution determine the properties of ultrafine wheat flour. Damage to starch granules was the most dominant factor influencing the final viscosity of flour/starch.

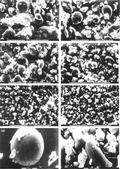

## 1 INTRODUCTION

Wheat (*Triticum aestivum* L.) is one of the most important food grains in the world and has a variety of food applications, including pocket breads, pan breads, noodles, steamed breads, and biscuits (Corke, Faubion, Seetheraman, & Wrigley, 2016). Wheat has been largely consumed by humans through the millennia with more than half of the world's population depending on wheat as their major source of protein and calories (Khan & Shewry, 2009). In general, the majority of wheat products are produced from wheat flour, and therefore, flour quality is critically important, particularly the flour/starch particle size distribution (Cauvain (2012)).

In order to process wheat grains, industry processes frequently employ different grinding techniques. During the wheat milling process, the endosperm (source of starch and proteins) is separated from the wheat kernel in order to obtain wheat flour with fine particle composition that can be used in many different processes. Importantly, the methods aimed at reducing the production particle size will significantly alter the composition of the flour. For example, size changes result in structural damages and changes to the granule profiles as a result of the frictional heat and mechanical energy during roller milling, which further affects the functional properties and the quality of flour‐containing food. In their work, Scanlon et al. (1988) observed that the water absorption capacity of wheat flour was significantly influenced by the flour/starch particle size (Scanlon, Dexter, & Biliaderis, 1988). Work by Kim and Shin (2014) evaluated the relationship between the flour particle size distribution and the pasting properties, revealing that the starch fraction peak intensity, final viscosities, and setback viscosities increased with decreasing particle size (Kim & Shin, 2014).

In addition to the wheat roll milling technique, superfine grinding is frequently used to decrease grain flour particle size and to improve the physicochemical properties of the fine powder, which influences the quality of the flour‐containing food (Drakos et al., 2017; Muttakin, Min, & Lee, 2015; Protonotariou, Batzaki, Yanniotis, & Mandala, 2016). Studies suggest that the superfine grinding techniques might improve the whole wheat flour quality by significantly reducing the powder particle size (Niu, Hou, Wang, & Chen, 2014). The superfine grinding process produces particles which are smaller than 40 μm (Chamayou & Dodds, 2007). Importantly, the process itself exerts particular effects on the properties of wheat flour, including an increase in water holding capacity (Protonotariou, Drakos, Evageliou, Ritzoulis, & Mandala, 2014) as well as changes in starch thermodynamic and dough rheological properties that can directly affect the preparation of fresh noodles (Niu et al., 2014).

Ultrafine grinding is another method used to decrease grain flour particle size, while jet milling is an important alternative to the ultrafine grinding technique that is used to significantly reduce the flour particle size. Jet milling is a fluid energy impact‐milling process that produces small particles (<10 μm) with a large surface area, high water absorption, and high solubility, which often result in more palatable foods. The objective of the present study was to investigate the effects of ultrafine grinding by jet milling on wheat flour, including the flour/starch particle size distribution, granule profiles, and the flour pasting properties. The overall goal of the study was to provide an important scientific contribution for the improvement of wheat flour quality.

## 2 MATERIALS AND METHODS

### 2.1 Material

The Zhengmai 9,023 cultivar samples were obtained from the Zhengzhou Haijia Food Company. The kernel hardness was 72%, and the protein content of the wheat samples was 14.8% (14% moisture basis, mb).

### 2.2 Preparation of straight‐grade flour by roller grinding

The wheat cultivar samples (water content: 11.7%) were cleaned, sieved, and handpicked to remove impurities, and then, the moisture was adjusted to 16% over the course of 24 hr. The straight‐grade flour (SGF) was obtained by roller milling of the wheat samples using Bühler mill MLU‐202 (Bühler Group) according to the AACC method 26‐21A Experimental Milling—Bühler Method for Hard Wheat (AACC, 2000), with the flour extraction rate of 70.2%.

### 2.3 Preparation of ultrafine flour power by jet milling

Five ultrafine flour samples (UFS) were obtained by jet milling the SGF on the QYF‐100 jet mill (Miyou Group Co., Ltd): UFS_1_, UFS_2_, UFS_3_, UFS_4_, and UFS_5_. During the ultrafine grinding process, the UFS particle size was reduced by collisions among the flour particles or between the flour particles and the inner wall of the grinding bowl at a low temperature (0°C–5°C) and high pressure (0.75–0.80 MPa). Overall, we obtained five different UFS particle size distributions by adjusting the rotation speed (from 3,000 rpm to 13,800 rpm) and number of grinding rounds (from one to three). The sample information is listed in Table 1.

**Table 1:** Particle size distributions of straight‐grade flour (SGF) and ultrafine flour samples (UFS)

Samples	Rotor speed (r/min)	Rounds of grinding	D_50_ (µm)
SGF	–	–	43.07 ± 0.03a
UFS_1_	3,000	Once	25.81 ± 0.03b
UFS_2_	7,800	Twice	21.11 ± 0.06c
UFS_3_	10,200	Once	15.22 ± 0.03d
UFS_4_	12,600	Three times	12.09 ± 0.07e
UFS_5_	13,800	Twice	10.15 ± 0.05f

Note

Data are presented as mean±standard deviation.

Different letters in the same line indicate significant differences (*p* < .05).

### 2.4 Flour particle size analysis

Different flour samples were thoroughly mixed, and the particle sizes were determined using a dry powder particle size Winner 3,000 detector (Jinan Winner Instruments Corporation). The instrumentation was equipped with a laser beam to detect the individual particles.

### 2.5 Scanning electron microscopy

Microstructure of the SGF and UFS was examined by AMRAY1000B SEM analysis (Amray, Inc). The samples were attached to aluminum specimen holders with double‐sided adhesive tape and coated with gold under a vacuum for observation. The microstructure of each sample was observed at 20 kV with a magnification of 800× and 3,000×.

### 2.6 Starch damage determination

Damaged starch content of the SGF and UFS was determined according to a previously published method (Medcalf & Gilles, 1965) using an assay kit and SDmatic starch damage analyzer (Chopin Technologies Corporation). The data for the damaged starch content were expressed as the percentage of flour weight on a dry basis.

### 2.7 The Hagberg–Perten falling number analysis

The Hagberg–Perten FN of SGF and UFS was estimated using a FN1800 instrument (Perten Instruments) according to the manufacturer's instructions. All determinations were performed at least three times, and the mean values were recorded.

### 2.8 Rapid viscosity analysis

The pasting properties of SGF and UFS were evaluated using a Rapid Viscosity Analyzer (RVA; Newport Scientific) according to the AACC method 61‐02. The pasting temperature (PT), peak viscosity (PV), trough viscosity (TV), final viscosity (FV), and the derivative parameters, including breakdown (BD = PV‐TV) and setback (SB = FV‐TR), were recorded. All determinations were performed at least three times, and the mean values were recorded.

### 2.9 Statistical analysis

The experimental results in this paper are reported as the mean of the results of two parallel experiments, and the Pearson correlation analysis was carried out to determine the relationship between different variables.

## 3 RESULTS AND DISCUSSION

### 3.1 Particle size

The particle size of SGF and UFS is summarized in Table 1 and expressed in terms of D_50_. D_50_ is the median diameter or median particle size and is commonly used to represent the average particle size of powders (Protonotariou et al., 2014). The range of particle size diameter (D_50_) was between 10.15 and 25.81 µm for UFS. The data suggest that jet milling resulted in a significant reduction of the average flour particle size with increasing milling speed and time.

Moreover, the results revealed that jet milling effectively transformed SGF into an ultrafine flour with particle sizes approaching 10 µm. As expected, the specific surface area was dramatically increased when the powder particle size was reduced. In their work, Zhang et al. (2012) reported that jet‐milled powder had a higher surface area compared with ground powder (Zhang et al., 2012).

The ultrafine grinding technique is an emerging technology that can produce a narrow and relatively homogenous particle size distribution with good surface properties, decent dispersibility in foods, and excellent absorption in the body (Tkacova & Stevulova, 1998; Zhao et al., 2009). Therefore, ultrafine grinding is particularly desirable for flour processing and food preparation for children and the elderly population (Li et al., 2012).

### 3.2 Scanning electron microscopy

**Figure 1:**
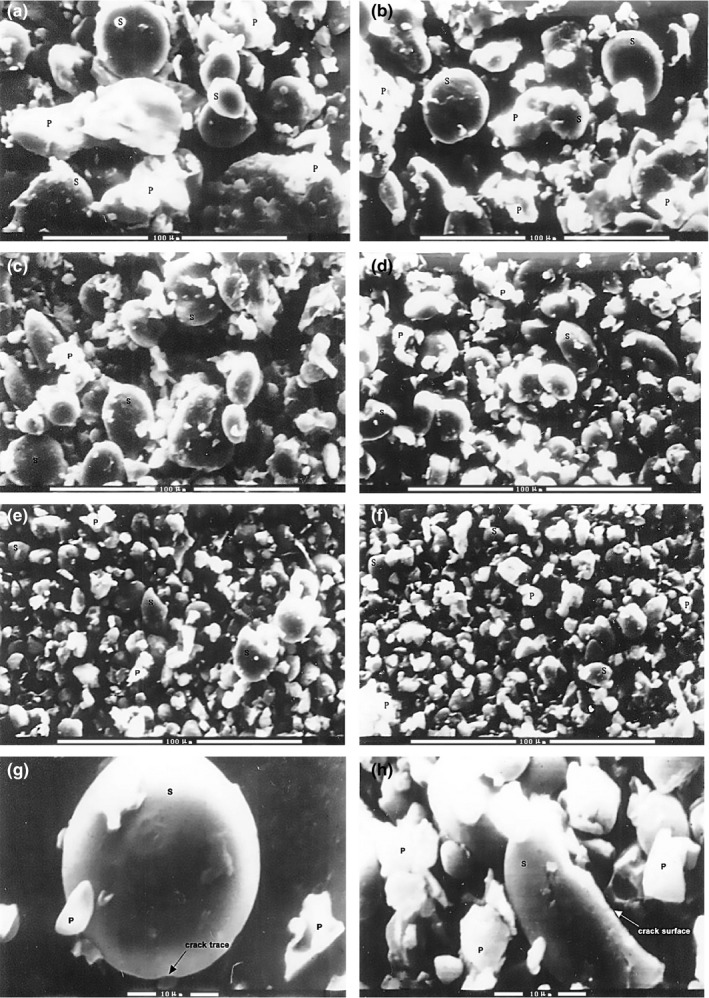
Scanning electron microscope (SEM) images of straight‐grade flour (SGF) and ultrafine flour samples (UFS). S, starch particle; P, protein fragment. (a) SGF, magnification 800×; (b) UFS_1_, magnification 800×; (c) UFS_2_, magnification 800×; (d) UFS_3_, magnification 800×; (e) UFS_4_, magnification 800×; (f) UFS_5_, magnification 800×; (g) UFS_5_, magnification 3,000×; and (h) UFS_5_, magnification 3,000×

The microstructure of SGF and UFS was observed using SEM (Figure 1). The particle sizes of the flour samples were significantly decreased as the rotation speed and grinding times increased. Compared with SGF, UFS appeared as very fine particles under the same magnification (×800). The starch in the SGF and UFS_1_ samples presented as round‐ and oval‐shaped particles with smooth surfaces (Figure 1g), and the particle size was largely dispersed (Figure 1a,b). In contrast, the starch particles in UFS_2_, UFS_3_, UFS_4,_ and UFS_5_ were more elongated in shape and appeared more homogeneous and compact compared with SGF and UFS_1_ (Figure 1c–f). Under the high pressure airflow inside the jet crushing cavity, flour/starch particle size was drastically reduced resulting in smaller, more solid particles and the structure of the flour/starch particles became significantly damaged. Many crack traces and cracked surfaces were also observed on the flour starch particles (Figure 1h). The ultrafine grinding process can result in highly damaged starch, which hydrates easily and is more susceptible to enzymatic hydrolysis (Sun et al., 2007). Furthermore, the ultrafine grinding technique can affect the thermal characteristics of flour/starch, as the starch granular structure changes. In agreement with our results, a previous study reported that the RVA FV of flour was strongly correlated with the degree of damage present in starch granules (Hasjim, Li, & Dhital, 2013).

### 3.3 Damaged starch content

**Figure 2:**
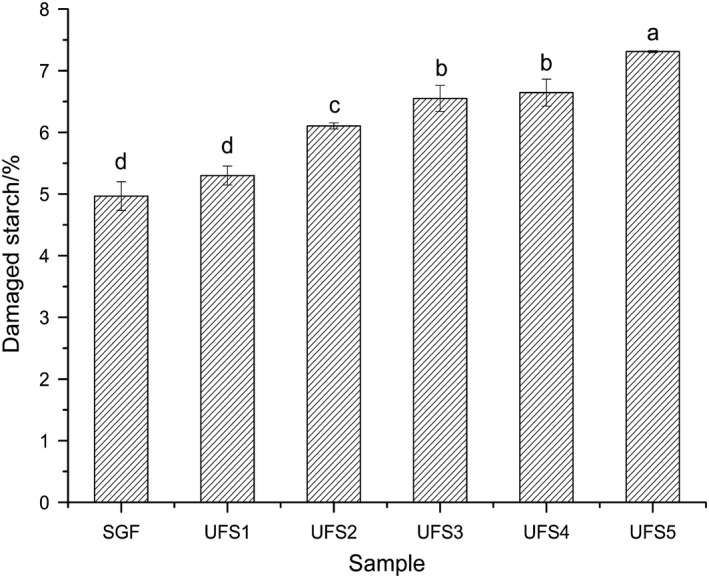
Damaged starch content of straight‐grade flour and ultrafine flour samples

The results indicate that starch damage increased significantly when the flour particle size was gradually decreased (Figure 2). The starch damage of UFS_5_ was the highest (7.31%). For SGF, damaged starch content was 47.1%. These results suggest that the amount of damaged starch was created in the flour by the jet‐milling process. Starch damage is an important and well‐recognized criterion of flour quality (Evers, Baker, & Stevens, 1984; Farrand, 1964; Hoseney, 1986; Salmon, Evers, & Harrison, 1990; Tipples, 1969) because it allows for easier hydration of the starch, making it more susceptible to enzymatic hydrolysis. Some studies suggest that a certain level of damaged starch content is beneficial because it increases the baking absorption and gassing power of the dough (Morrison & Tester, 1994; Rouilly, Rigal, & Gilbert, 2004). Overall, our results are in agreement with these studies, further suggesting that the jet‐milling process produces more damaged starch and in turn significantly improves the properties of the flour and the overall quality of wheat flour‐based products.

### 3.4 Falling number value

**Figure 3:**
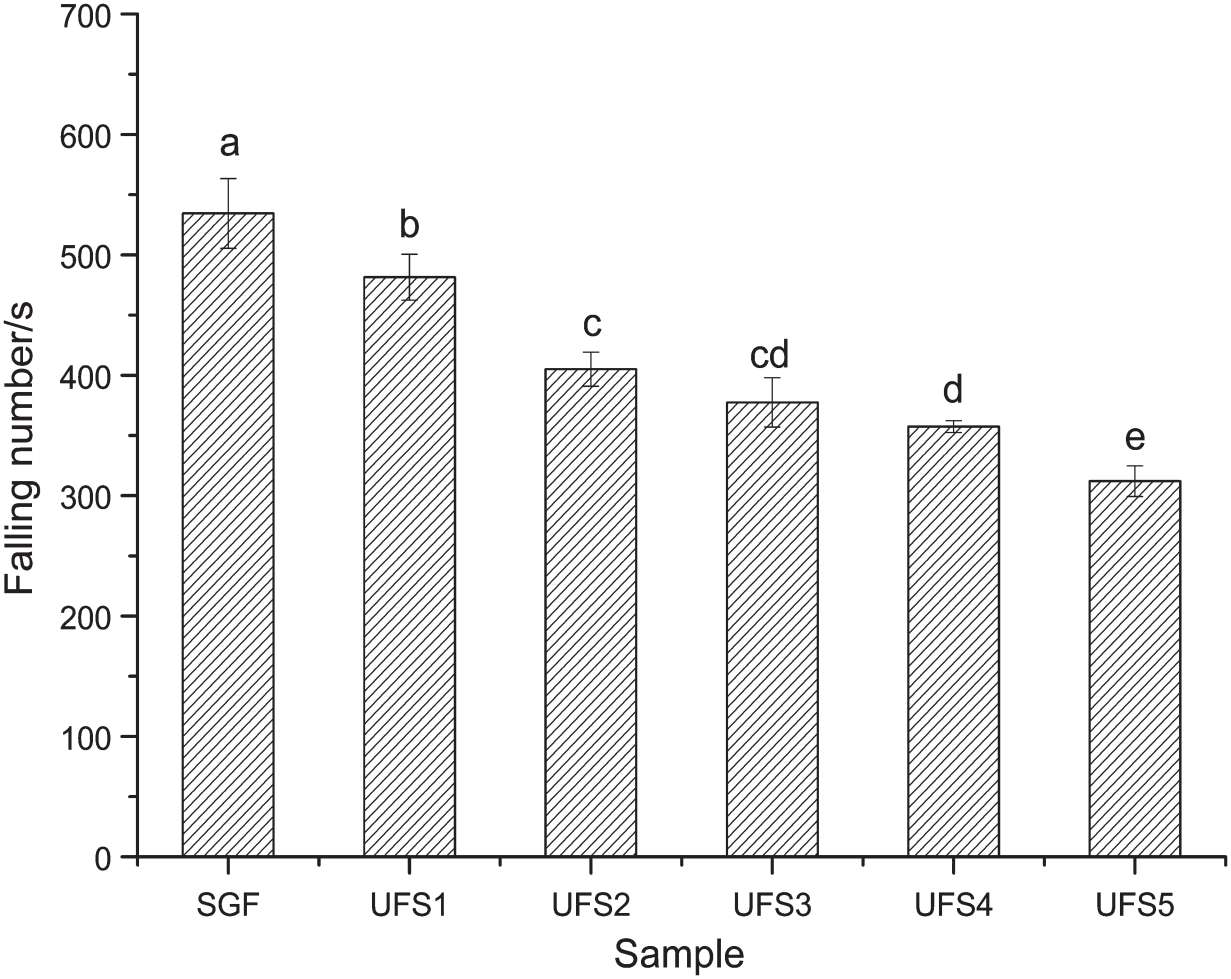
Falling number of straight‐grade flour and ultrafine flour samples

The FN apparatus is widely used for the rapid determination of grain alpha‐amylase activity, which is quantified in terms of the reduction in viscosity of a flour paste brought about by the action of an enzyme (Raschke, Taylor, & Taylor, 1995). High FN values indicate low alpha‐amylase activity. In the current study, we determined the FN value by the degree of starch damage, susceptibility of the starch to enzyme attack, and by the flour particle size distribution (Finney, 2001). The results revealed that the flour paste liquefied too rapidly when the flour particle size was significantly decreased. Compared with SGF, the FN of UFS_5_ decreased to 223 s (Figure 3). In general, the data suggest that the jet‐milling process decreased the flour/starch particle size, leading to an increase in damaged starch content, which together contributed to the lowering of the FN value.

### 3.5 Pasting properties

The pasting parameters of flour samples including PT, PV, TR, BD, FV, and SB are summarized in Table 2. We observed significant differences in the pasting parameters between different flour samples, suggesting that the flour pasting properties were largely affected by the flour particle size distribution. In contrast, we also observed that only the SGF showed a significantly lower PT compared with other jet‐milled flour samples. Specifically, PT is the temperature at which the flour/starch paste apparent viscosity starts to develop during heating in the RVA. Literature studies suggest that the PT of grain starch becomes reduced with increasing grinding times (Chen, Li, & Lu, 2003; Devi, Fibrianto, Torley, & Bhandari, 2009). In agreement with this research, another study identified a significant positive correlation between the PT of rice flour and flour particle size (Hasjim, Li, & Dhital, 2012). These results suggest that the reduction in PT of grain flour by milling is due to the disruption of flour particle size. In contrast, we observed significantly increased PT with jet‐milled flour. Overall, the results suggest that PT of the jet‐milled flour is influenced by a number of complex factors, not only a decrease in particle size. Other factors influencing PT include damaged starch granules, the disruption of the starch crystalline structure, and/or the degradation of starch molecules (Hasjim et al., 2013).

**Table 2:** Pasting parameters of straight‐grade flour (SGF) and ultrafine flour samples (UFS)

Sample	PT (°C)	PV ( RVU)	TV (RVU)	FV (RVU)	BD (RVU)	SB (RVU)
SGF	68.55 ± 0.01e	1582.00 ± 5.66b	1,041.00 ± 4.24b	2054.00 ± 5.66b	541.00 ± 1.41b	1,013.00 ± 1.41b
UFS_1_	86.25 ± 0.03d	1782.00 ± 4.24a	1,131.00 ± 1.41a	2,219.00 ± 9.90a	651.00 ± 2.83a	1,088.00 ± 8.49a
UFS_2_	87.85 ± 0.01a	1,357.00 ± 7.07c	872.00 ± 4.24c	1865.00 ± 1.41c	485.00 ± 2.83c	993.00 ± 2.83c
UFS_3_	87.10 ± 0.04c	1,180.00 ± 8.49d	762.00 ± 5.66d	1681.00 ± 9.90d	418.00 ± 2.83d	919.00 ± 4.24d
UFS_4_	87.71 ± 0.01b	980.00 ± 14.14e	591.00 ± 2.83e	1,363.00 ± 7.07e	389.00 ± 11.31e	772.00 ± 4.24e
UFS_5_	87.71 ± 0.02b	907.00 ± 8.49f	544.00 ± 5.66f	1,274.00 ± 4.24f	363.00 ± 2.83f	730.00 ± 1.41f

Note

Pasting temperature (PT) refers to the temperature at which the sample viscosity begins to increase after heating. Peak viscosity (PV) is the maximum viscosity value of starch paste heated before the sample is gelatinized. Trough viscosity (TV) is the minimum viscosity value of starch paste heated during cooling after it reaches peak viscosity. Final viscosity (FV) is the viscosity value of starch paste heated at the end of the test. BD represents breakdown, which is the difference between PV and TV. SB represents setback, which is the difference between FV and TV.

Data are presented as mean±standard deviation.

Different letters in the same line indicate significant differences (*p*< .05).

Furthermore, our data revealed that the PV and TV of jet‐milled flour decreased when the particle size decreased. These results suggest that the water holding capacity of the flour/starch was decreased after jet milling. In contrast, we also observed that the PV of UFS_1_ was significantly higher compared with the PV of the SGF sample. These results indicate that the water holding capacity of the flour/starch significantly increased when the flour particle size decreased to a certain degree (D_50_ reached approximately 25.0 µm); this could be explained by the increase in contact areas with water (Cornejo‐Villegas et al., 2013). Furthermore, the PV of the jet‐milled flour significantly decreased when the particle size decreased continuously. Studies suggest that the PV of flour/starch particles is a property of amylopectin, whereas the amylose–lipid complex can inhibit starch granule swelling and reduce the PV (Morrison, Tester, Snape, Law, & Gidley, 1993; Tester & Morrison, 1990). Overall, the data suggest that the amylose–lipid complex embedded in the wheat endosperm is likely released after the jet‐milling process.

BD viscosity reflects the stability of the flour/starch paste to withstand heating and shearing (Ilowefah et al., 2014). During the breakdown, the granules are disrupted, and consequently, linear molecules are released into the solution (Asmeda, Noorlaila, & Norziah, 2016). In this study, significant differences in BD viscosity were observed between SGF and UFS. Specifically, the BD viscosity of UFS_1_ was significantly higher compared with the SGF sample. In contrast, the BD viscosities of other jet‐milled flour samples were significantly lower compared with the SGF sample. These results suggest that all samples have different paste stabilities to withstand heating and shearing.

The correlation analysis results of flour/starch parameters are shown in Table 3. The FV was significantly correlated with flour/starch particle size, whereas a significant negative correlation was observed with the damaged starch content, which is in agreement with previously published results (Hasjim et al., 2013). Overall, these results suggest that damage to starch granules is the most dominant factor affecting the final viscosity of flour/starch.

**Table 3:** Correlation coefficients between different parameters of flour/starch

Property	Damaged starch (%)	Falling number (s)	PT (°C)	PV ( RVU)	TV (RVU)	BD (RVU)	FV (RVU)	SB (RVU)
D_50_ (µm)	−0.915**	0.944**	−0.900**	0.785**	0.815**	0.700*	0.787**	0.726**
Damaged starch	–	−0.967**	0.689*	−0.923**	−0.933**	−0.879**	−0.916**	−0.872**
Falling number	–	–	−0.759**	0.897**	0.912**	0.841**	0.891**	0.839**

* Represents significant differences at 0.05 level.

** Represents significant differences at 0.01 level.

## 4 CONCLUSION

Zhengmai 9,023 has one of the largest planting areas of any wheat variety in China (total of 7.7 million hectares). In this paper, we analyzed the properties of ultrafine flour from Zhengmai 9,023 samples. The data presented in this manuscript are the first to describe the effect of flour/starch granule profiles and particle size distribution on the FN value and pasting properties in ultrafine wheat flour. Our data clearly indicate that the particle size of flour/starch and its distribution are the dominant factors determining the properties of ultrafine wheat flour, including damaged starch content, FN value, and pasting properties. We discovered that the jet‐milling process significantly decreased the flour/starch particle size, which increased the damaged starch content; this may be directly responsible for changing the hydration characteristics and thermal properties of wheat flour. Our data suggested that when the particle size D_50_ was approximately 25.0 µm, all the pasting parameters of wheat flour were significantly increased in addition to changes in PT, which could be explained by the increase in the contact areas of the wheat flour with water. Furthermore, all the pasting parameters were significantly decreased when the particle size continuously decreased, which was also in addition to changes in PT. Importantly, what is not well‐understood and likely due to complex factors, is the decrease in wheat flour particle size and distribution, the damaged starch granules and content, the disruption of starch crystalline structure, and/or the degradation of starch molecules. Additional studies are warranted to address these knowledge gaps in the future.

In summary, this work serves as a foundation for further studies examining other properties of ultrafine wheat flour, including changes in wheat starch/protein composition and the improvement of the rheological properties of wheat flour starch/protein, dough, and overall food quality. Additional studies would be beneficial to fully explain the effects of ultrafine milling on the grinding properties of wheat grain and promote improvements in the quality of final wheat products.

## CONFLICT OF INTEREST

None.

## AUTHOR CONTRIBUTIONS

The authors are grateful to Ke Bian for critically reviewing this manuscript. The authors are pleased to acknowledge Yuling Yang, Jinyue Pang, Tingjing Zhang, and Mengmeng Li for improving the communication of manuscript.

## ETHICAL APPROVAL

This article does not contain any studies with human or animal subjects.
